# Nitrous Oxide

**Published:** 1866-02

**Authors:** Charles H. Shears

**Affiliations:** Sharon, Ct.


*Medical and Surgical Reporter.*

---

### NITROUS OXIDE.

EDITOR MEDICAL AND SURGICAL REPORTER:—Your reviewer, in his notice of "SANSOM ON CHLOROFORM," when speaking of *nitrous oxide* as an anæsthetic, says: "We ask, by the way, and would like to see answered, the query, what becomes, in the now increasing and successful use of nitrous oxide in surgery, of the wild excitement familiar to every student of chemistry."

The gas may be given so as to produce "wild excitement;" or it may be administered so as to produce perfect anæsthesia in about one minute. This difference in the effect of the gas, is found in the *quantity* given, and in the *manner* of administering it. Your chemist, who gives it to his student for amusement, uses a small bag of the capacity of two or three gallons, with a mouth-piece of small calibre and allows the air to mingle with the gas in the lungs.

Now, if instead of this miniature apparatus you use a six or eight gallon bag with a mouth-piece having a calibre of at least five-eighths of an inch, and having closed the nostrils of the patient, introduce the mouth-piece after an expiration, taking care that the lips are perfectly closed around it, so that no air shall enter, you will have no "wild excitement," but perfect anæsthesia.

CHARLES H. SHEARS, M. D.

*Sharon, Ct., Jan.* 22, 1866.

---

DR. BURTON, of Birmingham, Eng., has reported, in the last number of the *Dublin Quarterly Journal of Medical Science*, Nov., 1865, a case in which an artifical tooth and plate were swallowed during an attack of puerperal convulsions. It is stated that the patient recovered from the convulsions, but died four days afterward of gastritis.

